# Gas-assisted transformation of gold from *fcc* to the metastable 4H phase

**DOI:** 10.1038/s41467-019-14212-z

**Published:** 2020-01-28

**Authors:** Shaobo Han, Guang-Jie Xia, Chao Cai, Qi Wang, Yang-Gang Wang, Meng Gu, Jun Li

**Affiliations:** 1 Department of Materials Science and Engineering, Southern University of Science and Technology, No. 1088 Xueyuan Blvd, 518055 Shenzhen, Guangdong China; 2 Department of Chemistry, Southern University of Science and Technology, No. 1088 Xueyuan Blvd, 518055 Shenzhen, Guangdong China; 3 Theoretical Chemistry Center, Department of Chemistry, Tsinghua University, 100084 Beijing, China

**Keywords:** Nanoscale materials

## Abstract

The metastable hexagonal 4H-phase gold has recently attracted extensive interest due to its exceptional performance in catalysis. However, gold usually crystallizes to its lowest free energy structure called face-centered cubic *(fcc)*. The phase transformation from the stable *fcc* phase to the metastable 4H phase is thus of great significance in crystal phase engineering. Herein, we report this unusual phenomenon on a 4H gold nanorod template with the aid of CO gas and an electron beam. In situ transmission electron microscopy was used to directly visualize the interface propagation kinetics between the 4H-Au-nanorod and *fcc*-Au nanoparticle. Epitaxial growth was initiated at the contact interface, and then propagated to convert all parts of these *fcc* nanoparticles to 4H phase. Density functional theory calculations and ab initio molecular dynamics simulations show that the CO molecules can assist the Au diffusion process and promote the flexibility of Au particles during the epitaxial growth. The phase transformation was driven by the reduction of Gibbs free energy by eliminating the interface between *fcc* and 4H phases.

## Abstract (web summary)

Crystal phase engineering enables the growth of nanostructures with controlled crystal phases that show superior functional properties. Here, the authors find that CO gas-metal atom interactions combined with the electron beam can trigger phase transformations of precious metals at room temperature.

## Introduction

Noble-metal related nanostructures often show excellent catalytic properties due to their unique electronic structures that can be tuned by size, shape, defects, and crystal phases^1–4^. Especially polymorphs of the same composition often exhibit significantly different physical properties. Crystal phase engineering enable us to grow nanostructures with controlled crystal phases showing superior functional properties^5–7^. For example, gold nanostructures have been applied in catalysis, sensing, biomedicine, and surface-enhanced Raman scattering^1,8–11^. Normally gold crystalizes in face-centered-cubic (*fcc*) crystal structure, while other gold polytypes such as 2H and 4H-phase with AB and ABCB stacking order along close-packed [111] direction is less commonly found^12^. First principle calculations show that the formation energy of intrinsic, extrinsic and twin stacking fault energies along [111] direction in gold are one order of magnitude lower than other *fcc* metals. As a result, the cohesive energy difference between *fcc* and 4H phase is only as small as −2.2 meV, which is within the computation accuracy limit^13^. Controlled synthesis of 2H and 4H-phase gold nanostructures were recently proved possible using wet chemical method^14^. Experiments showed that the 4H-gold nanorods with ABCB stacking order presented well-developed surface plasmon resonance^5^. In addition, 4H/*fcc* gold nanorods can serve as template to epitaxially grow other metals with 4H phases, such as Rh, Pt, Pd, Os, Cu, Ir, Ag, and etc^15^. Superior mass electrocatalytic activity in oxygen evolution reaction, CO oxidation, ethanol oxidation catalysis was observed for those 4H/*fcc* phase structures^16,17^. High-energy electron beam driven phase transformation of gold from 2H-to-*fcc* crystal lattice structure has been revealed by earlier researchers^18^. Recently, researchers also proved that high pressure treatment led to the irreversible conversion from 4H-to-*fcc* phase^19^. All these findings are not surprising judging from thermodynamic stability and overall Gibbs energy of these two phases.

Here, by employing in situ transmission electron microscopy (TEM) and ab initio molecular dynamics simulations we demonstrate a solid-state *fcc*-to-4H phase transformation through epitaxial growth of *fcc*-Au nanoparticles on a 4H-Au nanorod template, which is activated by high energy electron beam and CO gas assistance. Our findings disclose that a combination of gas-metal atom interactions and electron beam can trigger phase transformations of precious metals at local nanoscale regions. In addition, certain rare crystal phases (such as 4H phase) of metals can be grown and fabricated using this template growth method in a gas environment.

## Results

### Structural characterization of the 4H-Au nanorods

**Fig. 1:**
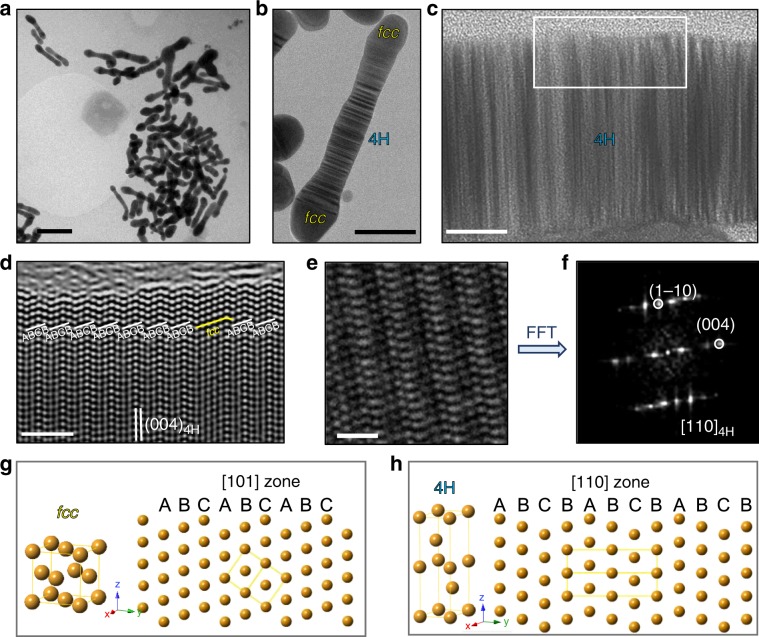
Microstructure of the Au nanorods. **a** Overall TEM image of as-synthesized Au nanorods; **b** larger magnification TEM of gold nanorod showing stacking fault contrast in the 4H phase region in the middle; **c** HRTEM showing the stacking fault contrast in the 4H phase; **d** HRTEM at selected area in the white rectangle region in **c** showing ABCB stacking order in 4H phase in conjunction with *fcc* twins; **e** HRTEM image at other region also showing 4H repeating order and its FFT in [110]_4H_ zone in **f**; comparison of the atomic model of *fcc* Au structure (**g**) and 4H phase (**h**). The scale bar in panel **a**–**e** is 200 nm, 50 nm, 5 nm, 2 nm, 1 nm, respectively.

As shown by the TEM image in Fig. 1a, the as-synthesized gold nanorods are about 300 nm long and 20–25 nm in diameter. The Au nanorods contain two phases-a majority 4H phase bridged by *fcc*-twins as shown by Fig. 1b. In addition, the two ends are *fcc*-phase, while the middle part is mostly 4H phase as proved by the ordered-stacking fault contrast in Fig. 1b, c. The atomic structure of the middle part is examined by high resolution TEM (HRTEM) imaging and fast fourier transform (FFT) analysis in Fig. 1d–f. As clearly shown by the *fcc* and 4H models in Fig. 1g, h, the atoms in *fcc* Au crystals are arranged by ABC stacking order, while the atoms in 4H Au crystals are arranged by ABCB stacking order. Comparing the atomic models with the atomic-resolution HRTEM image in Fig. 1d, e, middle portion of the nanorods is composed of 4H phase as labeled by the white lines bridged by *fcc*-twins labeled by the yellow lines. The FFT analysis in Fig. 1f also shows the characteristic 4H ordering diffraction pattern in [110]_4H_ zone with (1–10) and (004) surface planes circled in white.

### Orientation relationship between *fcc* Au nanoparticles and 4H-nanorods

**Fig. 2:**
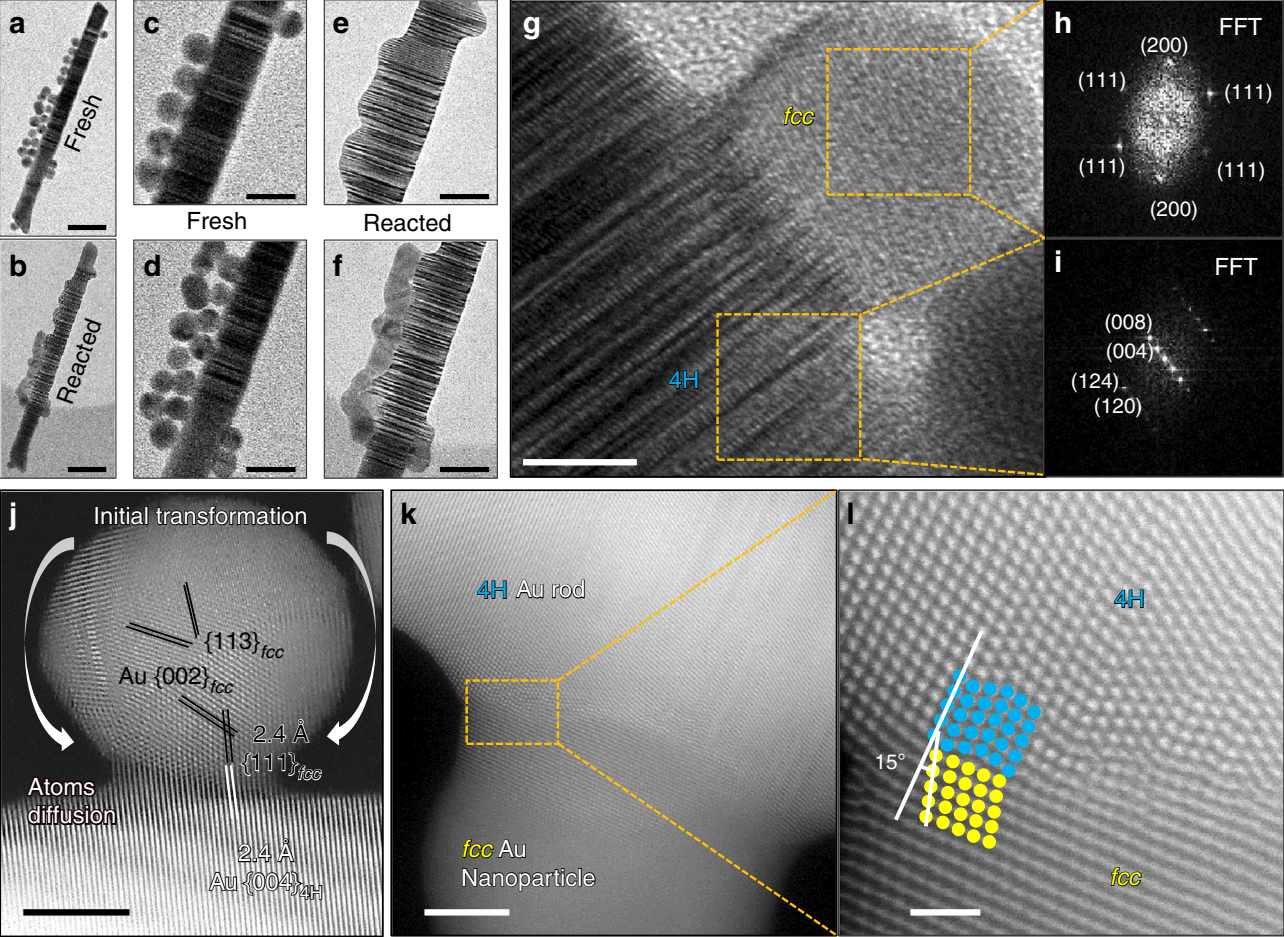
Orientation relationship between the fcc nanoparticles and 4H rod. **a** TEM image of original fresh *fcc* gold nanoparticles on a 4H/*fcc* gold nanorod; **b** TEM image of the same region in **a** after reaction (the scale bar in **a**, **b** corresponds to 100 nm); magnified TEM image of the top/bottom portion of the fresh sample (**c**, **d**) and reacted sample (**e**, **f**) (the scale bar in **c**–**f** is 20 nm); **g** high resolution TEM image showing the orientation relationship of the Au nanoparticle and 4H nanorod during reaction; **h** FFT of the square region at the *fcc*-Au nanoparticle **h**; **i** FFT of the square region at the 4H rod; **j**–**l** atomic scale Z-contrast scanning TEM imaging of the interface between *fcc* gold nanoparticle and 4H-gold rod at the early stage of phase transformation; the {111} crystal planes of the *fcc* Au nanoparticle are well aligned with the (004) crystal planes of the 4H rod with a small 15° tilt angle (the scale bar in **g**, **j**–**k** is 5 nm; the scale bar in **l** is 1 nm).

We put a few Au nanoparticles on the surface of the 4H/*fcc* nanorods as shown in Fig. 2a. Then we inputted CO gas into the TEM chamber and shined electron beam with 300 keV energy and a dose rate of 4000 e Å^−2^ s^−1^ on the particles and nanorods for about 5 min at room temperature without additional resistive heating. The same region after reaction evolved to be different morphology as shown in Fig. 2b. The higher-magnification images of the top and bottom parts of the particles and 4H nanorods are shown in Fig. 2c, d. The corresponding reacted TEM images of the same regions are shown in Fig. 2e, f. The ordered-stacking fault-like contrast in the TEM image in Figs. 1c,  2e, f is the signature of the 4H phase. At the initial stage in Fig. 2a, c, d, the Au particles are spherical sitting on top of the underlying Au-rod. The joining of the *fcc* Au particles and 4H-Au rod started by Au atom diffusion in CO gas with electron beam irradiation as shown by combined HRTEM and high-angle-annular-dark-field (HAADF) Z-contrast imaging in Fig. 2g–l.

A detailed analysis of the interfacial orientation relationship was carried out using high resolution TEM and related FFT analysis in Fig. 2g–i. As revealed clearly, the phase of the Au rod can be clearly identified as 4H crystal by the indexed FFT diffraction pattern in Fig. 2i, while the *fcc*-Au particle exhibited clearly *fcc* symmetry as shown by Fig. 2h. A close inspection of the interface by comparing FFT diffraction patterns of the two parts revealed that the {111} crystal planes of the *fcc*-Au particle aligned well with (004) crystal planes of the 4H rod but with a small tilt angle of ~15°. In addition, using atomic scale Z-contrast imaging, we verified the orientation relationship. As found by Fig. 2j, We can index these Au particles to be *fcc* crystal with {111}&{113}&{002} crystal planes in Fig. 2j. The {111}_*fcc*_ crystal planes in the Au nanoparticle aligned well with the (004)_4H_ crystal planes in the Au-rod, forming epitaxial interface as shown in Fig. 2j.

In addition, we captured the reacted intermediate state using high resolution Z-contrast imaging, where the *fcc*-Au nanoparticle and 4H rod are simultaneously in zone axis shown in Fig. 2k, l. The atomic columns of 4H-Au rod are circled in blue and the atomic columns of fcc-Au nanoparticle are circled in yellow in Fig. 2k, l. In-depth atomic column analysis in Fig. 2l also revealed the epitaxial interface with {111}_*fcc*_ aligned with (004)_4H_ surface planes with a 15° tilt angle, which is consistent with the FFT analysis in Fig. 2h–i. The interface became epitaxial through the joining of the {111}_*fcc*_ & (004)_4H_ crystal planes, which is quite similar to the oriented attachment growth as reported by Li et al.^20^, where two particles with aligned orientations facilitate the epitaxial growth. The oriented attachment can take place in either liquid or gas environment with van der Waals forces as driving forces. As observed, the *fcc*-Au particles firstly sintered on the 4H-phase nanorod through aligning its {111}_*fcc*_ set of crystal planes with the (004)_4H_ surface planes of 4H-rod and transformed to 4H phase in the end.

### In situ TEM characterization of the phase transformation

**Fig. 3:**
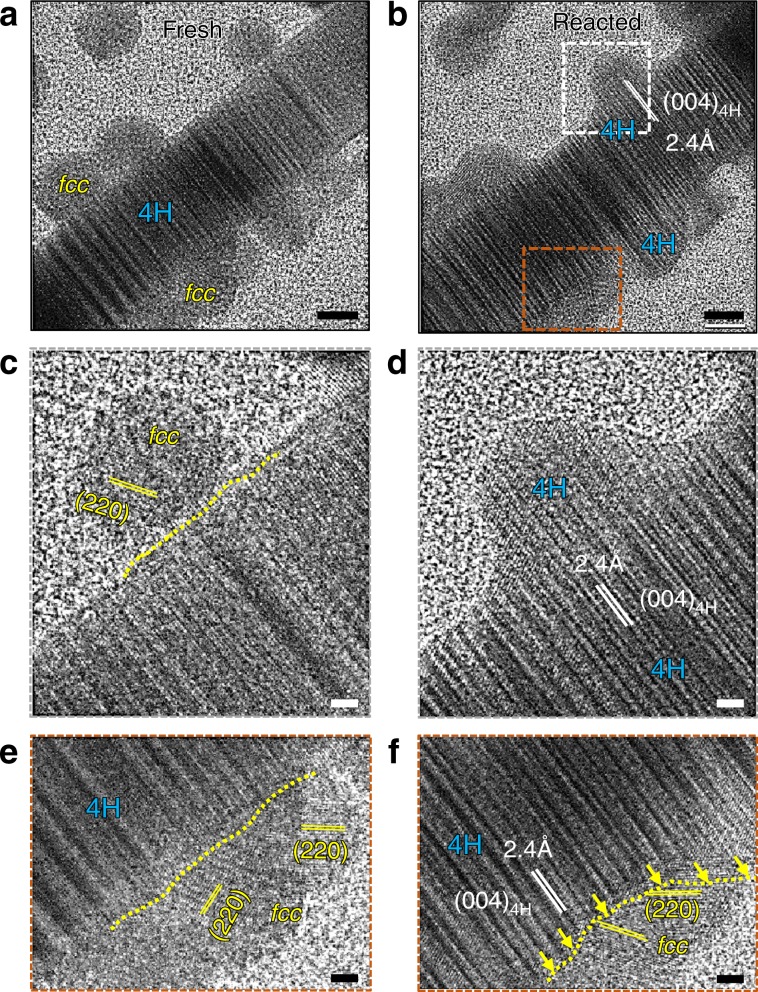
Phase transformation details of the initial and the final states. **a** HRTEM showing the original fresh (**a**, **c**, **e**) and reacted (**b**, **d**, **f**) sample of the same region. The yellow lines in **c**, **e**, **f** labels the boundary between 4H and fcc phase; the yellow arrows in **f** labels the propagation direction of the phase conversion process; please note that panel **c**, **d**; **e**, **f** compares the initial and intermediate state of the phase transformation process at the atomic scale. (the scale bar in **a**, **b** is 5 nm and the scale bar in **c**–**f** is 1 nm).

Careful examination of the *fcc*-to-4H conversion at room temperature is illustrated in Fig. 3. The fresh *fcc-*Au nanoparticles were arranged on the surface of 4H/*fcc* Au nanorod in Fig. 3a. The reacted morphology of the same region is shown in Fig. 3b. The morphology of the particle labeled by the white square before and after reaction is magnified in Fig. 3c, d. The lattice of this particle in Fig. 3c corresponds to the {220} crystal planes of *fcc*-Au. Please note that the {220} planes of the *fcc* region misaligned with the 4H-phase interface at the fresh state as shown in Fig. 3c. After reaction in Fig. 3d, this particle fully transformed to 4H-phase that is in perfect epitaxy with the underlying 4H-nanorod. During the *fcc*-to-4H transformation, the gold nanoparticle must align its orientation to match the underlying 4H-Au rod and form perfect epitaxy. In addition, the magnified HRTEM view of the brown rectangle region in Fig. 3b before and after reaction is shown in Fig. 3e, f, respectively. The interfaces between the 4H and *fcc* phase are labeled by yellow dashed lines in Fig. 3e, f. As shown by the yellow arrows in Fig. 3f, the 4H-to-*fcc* transformation reaction front progressively pushed forward to the *fcc* Au particles region.

**Fig. 4:**
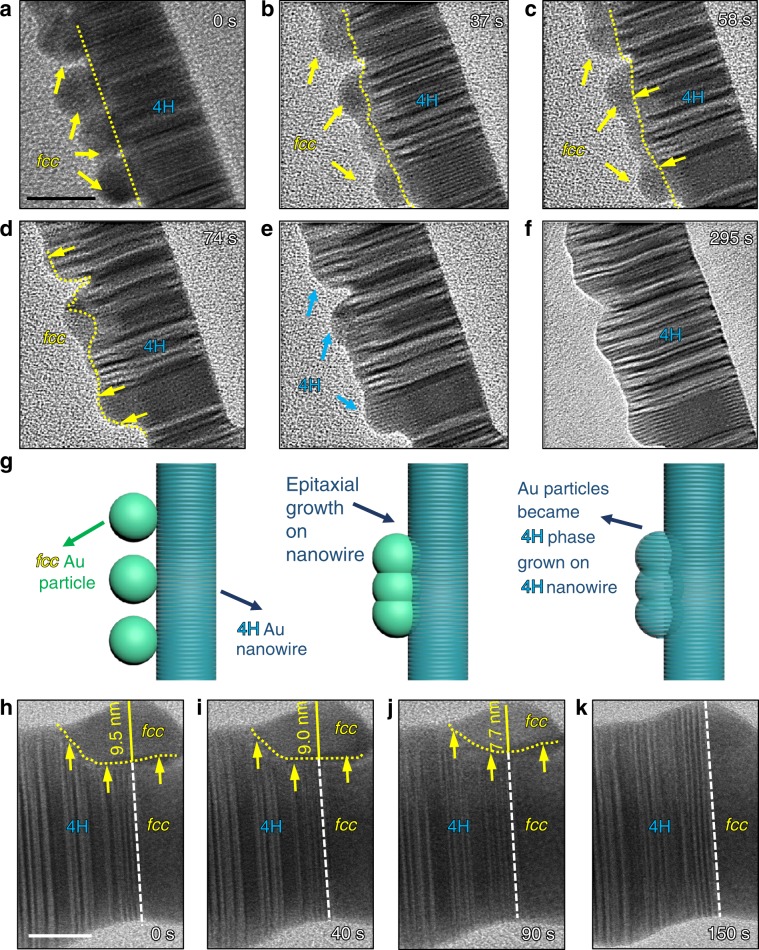
In situ TEM observation of the phase transformation. **a**–**f** Atomic structure of the interface at different reaction time; the observation is performed at 300 kV with electron beam dosage of 4000 e^−^ Å^−2^ s^−1^ in 1 mbar of CO gas (the scale bar in panel a is 20 nm and can apply to panel **b**–**f**; **g** schematic drawing showing the *fcc*-to-4H phase transformation of the Au nanoparticles on 4H region of the rod; **h**–**k** in situ TEM observation of the phase transformation of a *fcc*–Au nanoparticle siting on the boundary between the 4H region and one *fcc*-end of the nanorod; experiments were performed at room temperature at 300 kV with electron dose of 2000 e Å^−2^ s^−1^ in 1 mbar of CO gas (the scale bar in **h** is 10 nm and can be applied to panel **i**–**k**).

Supplementary Movie 1 and Fig. 4 illustrate the dynamic transformation process from *fcc* to 4H Au by observing the Au particles on the surface of the 4H nanorod using in situ TEM at room temperature. The morphology of the fresh sample is characterized by a few Au nanoparticles on the 4H-Au nanorod in Fig. 4a. These particles sintered on the 4H wire and formed aggregates as shown by the images in Fig. 4b, c. The interface between the *fcc* and 4H phases is labeled by dashed yellow lines. The interface progressively pushed forward into the *fcc* phase region as shown by the yellow arrows in Fig. 4c, d. At 148 s, the *fcc* particles transformed fully to 4H phase. After another few minutes at 295 s, the as-formed 4H phase is stable under the intense electron beam.

As illustrated by the schematic drawings in Fig. 4g, firstly the Au atoms from *fcc*-Au nanoparticles were activated by the high-energy electron beam and diffused to the interface region in CO gas. The Au diffusions resulted in sintering of Au particles to form interconnected aggregates and the *fcc*-to-4H phase transformation took place at the interface between these two phases. The 4H-Au phase progressively grew larger in CO gas under the electron beam irradiation and the interface pushed forward by consuming the *fcc-*Au phase.

Another interesting observation was shown in the Fig. 4h–k, where a *fcc*-Au nanoparticle sits on the boundary region between the 4H region and one *fcc*-end of the rod. Corresponding movie showing the phase transformation process can be found in the Supplementary Movie 1. As found, the Au part sitting on the 4H region progressively evolved to 4H phase, while the other part sitting on top of the fcc-end of the rod retained *fcc* crystal. Therefore, the phase transformation process is closely related to the epitaxial growth mechanism, where a reduction of the interfacial energy between these two phases provides the driving force.

In contrast, control experiments showed that no reaction took place under the same electron dosage in vacuum or Ar-gas environment at room temperature. Therefore, the CO bonding with Au atoms must effectively facilitate Au diffusion and realignment of *fcc* lattice to form ordered-stacking faults in the newly formed 4H phase. Previous study found that CO gas has strong bond with Au atoms and make the diffusion or jump of Au atom much easier^21,22^. Dynamic gold single atoms can break away from maternal nanoparticle during reaction due to the strong interaction between CO gas and gold atoms^9^. In our study, we unambiguously resolved the CO-assisted 4H phase formation from original *fcc* crystal lattice of gold. The interfacial energy is significantly lowered after the phase transformation by forming coherent interfaces with underlying 4H-nanorod. The minimization of total Gibbs energy of *fcc*-nanoparticles on 4H-nanorod led to cohesive epitaxial growth and formation of 4H phase in the Au nanoparticles. As shown by Batson et al.^23^, coalescence of Au nanoparticles with 1–2 nm in size can take place by attractive interparticle forces resulting from the coupling of surface plasmons in response to the passage of electron beam. In our experiment, this effect may lead to the observed merging of the *fcc* Au nanoparticle on the 4H wire in Supplementary Fig. 8, where the plasmonic responses could also promote the Au diffusion at the interface.

### Theoretical simulations on the *fcc*-to-4H transformation mechanism

To figure out the phase transformation mechanism, theoretical calculations based on the density functional theory (DFT) and ab initio molecular dynamics (AIMD) were performed. To model the epitaxial Au diffusion, the Au *fcc* {111} pillar modeled with 2 × 2 unit cell of four layers (16 Au atoms per layer) is sintered on a 4-layer 4H substrate (36 Au atoms per layer). This model corresponds to the experimental observation shown in Fig. 2j–l, where the {111} surfaces of fcc-nanoparticles are aligned with the (004) surfaces of 4H rod. We neglected the local structure rearrangement and defects at the 4H/*fcc* interface for simplicity and focused on studying the two crucial factors for the transformation process, i.e., the epitaxial Au diffusion and Au layer slide in *fcc* nanoparticle. We considered two possible facts for describing the promotion effect from electron beam. On one side, the kinetic energy from the electron beam is helpful to overcome the kinetic barriers for the transformation process. On the other side, our sample is possible to be affected by extra electrons, which may be directly from the electron beam or from the decay of surface plasmon excited by the electron beam^24–31^. We, therefore, include extra electrons into our system to explore the possible promotion effect. See Supplementary Figs. 1–4 for details of our model.

**Fig. 5:**
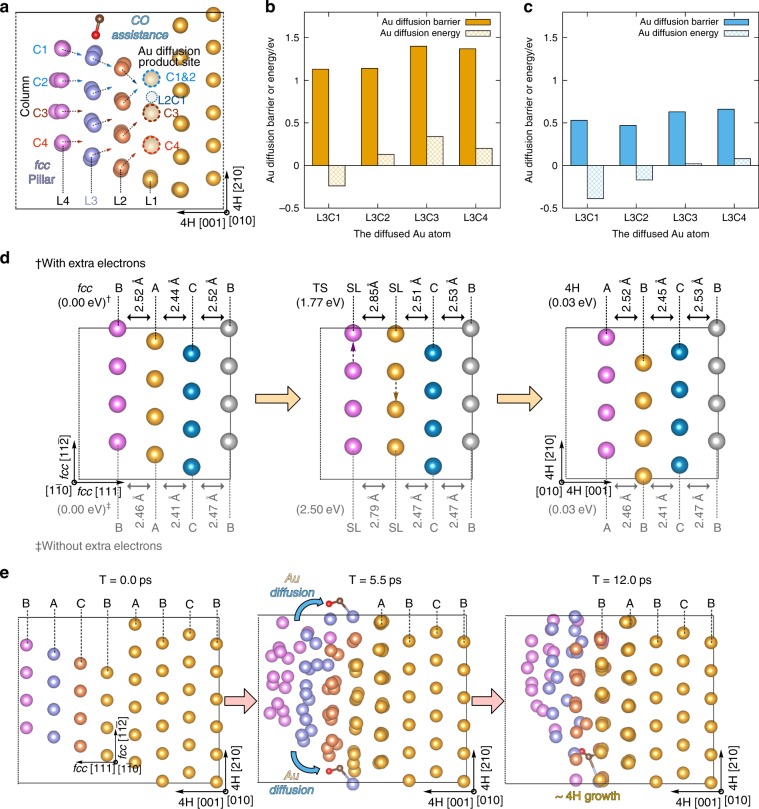
Theoretical studies on *fcc*-to-4H transformation. All atoms expect the CO molecule in the figure are Au atoms. **a** Label of the Au diffusion site and the Au diffusion direction from *fcc* pillars to L1. For brevity the rightest two 4H layers are not shown, and the full atomic model is shown in Supplementary Fig. 1b, c. The active energy and reaction energy of Au diffused from L3, the typical *fcc* layer, to L1, the 4H substrate: **b** normal reaction; **c** reaction with one CO binding on the diffused Au. **d** Au layer slide assisted by extra electrons. The black numbers are the results calculated with extra electrons, which distribution is shown in Supplementary Fig. 4; the gray numbers are that without extra electrons. **e** Typical structures in AIMD trajectories with one CO and extra electrons.

During the optimization, the Au atoms of the *fcc* pillar have some displacement from its ideal position due to the aperiodicity. As shown in Fig. 5a, it is possible to diffuse one Au on the *fcc* layers to the 4H substrate (L1). Here the diffusion of the 12 Au atoms from the three *fcc* layers (L4/L3/L2) are all studied with typical reaction structures shown in Supplementary Fig. 2 and active energy (*E*_a_) and reaction energy (Δ*E*) shown in Supplementary Table 3. The *E*_a_ and Δ*E* of the Au diffusion from the typical *fcc* layer L3 to 4H substrate without and with CO assistance are shown in Fig. 5b, c, respectively. Without CO the Au diffusion barriers are high and all over 1 eV. When the diffused Au is bonded to CO forming the Au–CO complex, its barrier is dramatically reduced and the diffusion energy also becomes slightly more favorable, as shown in Fig. 5c. This CO assistance effect is valid not only for L3 Au atoms but also for L2 and L4 Au atoms, which is listed in Supplementary Table 3. The Au–CO coordinate bond can pull the Au atom out from the nanoparticle and weaken its interaction with other Au atoms, which helps Au diffusion to 4H substrate. Such effect is also observed in the previous theoretical study^21^. Extra electrons only contribute to slight drops on barrier and reaction energy, which is also shown in Supplementary Table 3. Although in experiment this Au epitaxial diffusion could also take place on different surfaces at the interface, the transformation process and its chemical picture are clear: by forming Au–CO complex, the CO could dramatically assist Au diffusion to 4H substrate.

For the *fcc*-to-4H transformation of the Au atoms inside *fcc* nanoparticles, where CO cannot approach, it could take place by layer sliding in Supplementary Movie 1. To study this, the model consisting four Au *fcc* layers is built for simplification, as shown in Fig. 5d. With some floating up on the average layer spacing, there is an overall slide of the Au *fcc* layer, resulting in the transformation of *fcc* ABC packing to the 4H BCBA packing. When extra electrons are introduced, as shown in Supplementary Fig. 4b, they distribute on the Au–Au anti-bonding orbitals of both the outer and inner Au layers, which weakens the Au layer–layer interaction. Comparing to the neutral case, these extra electrons could reduce Au *fcc* layer sliding barrier dramatically by 0.73 eV.

At last, the dynamic property of this transformation was investigated. AIMD was done at the room temperature (300 K) with a time step of 1 fs and lasts for 12 ps. The corresponding AIMD movie showing the detailed trajectory of atoms is shown in Supplementary Movie 2. During the AIMD calculation, no collapse of *fcc* pillar is observed in the system without CO, as shown in Supplementary Fig. 5. However, even when only one CO is adopted on the surface of Au *fcc* pillar, the CO-bonded Au will soon be diffused to the 4H substrate within 3 ps, as shown in Fig. 5e. The process is quite similar to the study of Au diffusion in Fig. 5a–c. With the effect of thermal energy at this temperature, the Au–CO complex will also pull other Au out leading to the overall collapse of the *fcc* pillar at 5.5 ps. Interestingly, besides the fast movement of Au–CO complex, the CO itself can diffuse on Au surface sometimes and form new Au–CO complex, further facilitating the transformation process. The diffusion of CO itself was not observed in our previous MD simulations^9^ as the Au cluster was largely reconstructed under high CO coverage. Without extra electrons in Supplementary Fig. 6a, in AIMD the Au–CO complex can also diffuse to 4H substrate with the *fcc* pillar collapse taken place, but at 12 ps the newly formed 4H layers on the substrate are still in balance rather than forming a basically ordered 4H layer. These AIMD calculations clearly demonstrate the CO is the key factor for Au diffusion while the extra electrons could be helpful to construct new Au 4H layers. Based on our simulations, we noted that CO could not only assist Au diffusion at the interface, but also increase the flexibility of the Au particles, which may promote the layer-sliding during the phase transformation. The Supplementary Movies 2–5 showing the detailed atomic diffusion trajectories of AIMD are also provided in the supporting information.

**Fig. 6:**
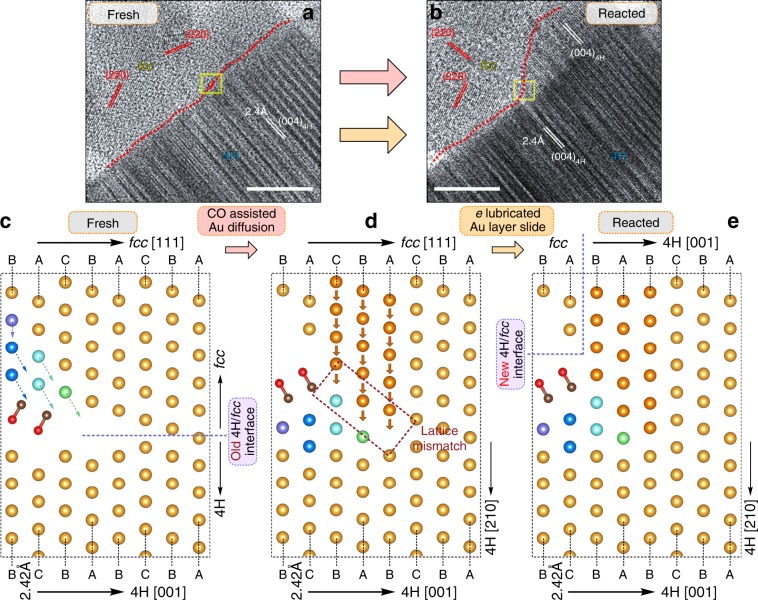
Schematic reaction mechanism of the epitaxial growth. HRTEM image showing the propagation between the *fcc* crystal lattices of the nanoparticles and 4H phase of the rod region before (**a**) and after (**b**) reaction. Both the scale bars in panel **a**–**b** corresponds to 5 nm. Please note that the interface between the 4H and *fcc* phases are labeled by the red dashed lines. **c**–**e** The atomic models schematically showing the *fcc*-to-4H transformation process.

Comprehensively speaking, the *fcc* to 4H transformation can be illustrated in Fig. 6. Figure 6a, b examined the lattices before and after reaction at the same interface, in which the Au-*fcc* nanoparticles partially transformed to 4H phase as indicated by the high-density ordered-stacking-fault contrast. The transformation is based on an epitaxial growth mechanism. Initially, there is a gap between the nanoparticle and 4H-Au rod. After the reaction started, the diffused Au atoms firstly filled the gap. Then, the reaction front push forward to transform most of the fcc nanoparticle to 4H phase. The atomic models of the *fcc*-to-4H transformation mechanism is visually shown in Fig. 6c–e. The reaction is initially activated by the CO assisted Au short-range atom diffusion and local atoms reconstruction. After the gap between the interface of 4H and *fcc* is nearly patched by Au diffusion, the layer sliding can take place, which eliminate the lattice mismatch. Newly formed 4H phase can also induce Au-layer sliding inside the *fcc* nanoparticle, which results in *fcc*-to-4H transformation of inner part of the *fcc* nanoparticle. The Au-layer sliding is possibly promoted by three factors, (i) the increased thermal energy from electron beam (the beam heating effect on the temperature rise at varies voltages and dose rates are shown in Supplementary Table 4; the estimation method see Supplementary Method for detail); (ii) the increased flexibility of gold due to CO adsorption; (iii) the possible extra electrons from electron beam or the locally electron-rich environment^32–34^. As shown by Supplementary Fig. 8, at lower voltage at 80 kV with a dose of 500 e Å^−2^ s^−1^, we can still observe the phase transformation process clearly. The corresponding Supplementary Movie 6 showed this process vividly. In a word, the synergetic effect of CO and electron beam makes the *fcc*/4H interface advanced continuously. Finally, we noted that the thermodynamic driving force of the *fcc*-to-4H transformation comes from the relatively higher *fcc* surface energy comparing to 4H. A competition of the lower bulk energy of *fcc* phase and the lower 4H surface energy results in a critical size that is calculated to be around 9.5 nm, which is also consistent with the experimental observation (See Supplementary Discussion for detail). The phase transformation processes halted due to the bigger size of the Au nanoparticle are shown in Supplementary Figs. 9, 10. The corresponding Supplementary Movies 7, 8 showed this process vividly.

In conclusion, a phase transformation from *fcc* to 4H phase for gold nanoparticles on a 4H-gold nanorod template can be driven by electron beam in CO gas environment. The epitaxial conversion process of *fcc*-gold nanoparticles to 4H phase was directly visualized using atomic scale in situ TEM at varies conditions. Density functional theory calculation and ab-initio molecular dynamics simulation show the CO molecules can assist the Au diffusion process and promote the flexibility of Au particle during the epitaxial growth. The counter-intuitive phase transformation was perfectly explained by the overall Gibbs free energy reduction due to the interface energy elimination. The present work suggests that the bonding and epitaxial growth of different metal nanostructures may become reality using gas-metal atom interactions controlled precisely at nanoscale using electron beam. Gas and electron beam-activated phase conversion may be largely applied to the microscopic phase engineering for future devices and catalysis.

## Methods

### Sample preparation

In a Schlenk tube, HAuCl_4_ (40 mg dissolved in HCl) were added in hexane (4 ml). This solution was then injected into oleylamine (20 ml) at 80°C. The hexane was evaporated in low vacuum. After the solution became transparent, 1,2-dichloropropane (0.5 ml) was injected into the solution. After another 1 min stirring at 300 rpm, the solution was hold at 80 °C for 6 h. Then, the red brown precipitation was collected using centrifugation and washed by isopropanol and hexane solution (with volume ratio of 3:1) via centrifugation at 10,000 rpm for 5 min. After centrifugation, the liquid layers were discarded and only the precipitation was collected. The above washing procedure were repeated 5 times. The final products were dispersed in ethanol and dropped onto a TEM grid. The TEM grid was then left to dry for 3 h. In addition, we baked the TEM grid with infrared light for 15–25 min to evaporate the residue organic molecules on the Au surface. No organic residues were observed in the TEM analysis and FTIR test.

### In situ TEM

In situ TEM was performed using aberration-corrected FEI Environmental TEM (ETEM) at varies acceleration voltage and electron dose, including 80 kV (Dose = 500 e Å^−2^ s^−1^), 300 kV (Dose = 4000 e Å^−2^ s^−1^), and 300 kV (Dose = 1000 e Å^−2^ s^−1^). The ETEM used differential pumping system, which allows us to input 1mbar of CO gas into the sample chamber. The images are acquired using FEI-Ceta-II fast camera with 4k × 4k pixels.

### Theoretical modeling

The density functional theory (DFT) calculation is implemented within the framework of periodic boundary condition using the VASP code^35^. Projected augmented wave method^36^ is used to describe the ion–core electron interaction, while the exchange and correlation interactions are described by PBE functional^37^. Dispersion interactions are involved by applying D3 corrections^37^. As this system is considerably large, the Gamma point k-point sampling from is used with the kinetic energy cutoff of 400 eV. Further test on k-point sampling and kinetic energy cutoff are done and shown in Supplementary Tables 1, 2. Both the increment of cutoff and K-point only contribute to a tiny change on the relative energy, which are all smaller than 0.013 eV. The convergence criterion for total energies is 10^−6^ eV with the Methfessel-Paxton smearing scheme of 0.2 eV, while the forces acting on the atoms were smaller than 0.02 eV Å^−1^ for geometry optimization. Climbing image nudged elastic band (CI-NEB)^38^ and the dimer method^39^ are both used to calculate the transition states (TS), and the forces acting on the atoms in TS are smaller than 0.05 eV Å^−1^. When extra electrons are introduced, although a dose rate of electron bean is 4000 e Å^−2^ s^−1^, considering the Au is a conductor and our calculation model is quite small comparing to the actual Au nanorod and nanoparticles, only few extra electrons are added into the system: 8 extra electrons in the study of diffusion barriers and energies; 16 electrons in the AIMD study. The movies of the AIMD trajectories in detail are also provided as Supplementary Movies 2–5, and the typical structures are shown in Fig. 5e, Supplementary Figs. 5, 6.

## Supplementary information

Supplementary Information

Description of Additional Supplementary Files

Supplementary Movie 1

Supplementary Movie 2

Supplementary Movie 3

Supplementary Movie 4

Supplementary Movie 5

Supplementary Movie 6

Supplementary Movie 7

Supplementary Movie 8

## Data Availability

The data presented in this manuscript is available from the corresponding author on reasonable request.
