# Supplementary material for: Gas-assisted transformation of gold from fcc to the metastable 4H phase
Source: Nat Commun. 2020 Jan 28;11:552. doi: 10.1038/s41467-019-14212-z (PMC6987310; doi:10.1038/s41467-019-14212-z)
Supplement: Supplementary file 1 — Supplementary Information [file 41467_2019_14212_MOESM1_ESM.pdf]

Gas-assisted Transformation of Gold from *fcc* to the Metastable 4H Phase

Han et al.

Supplementary Figures

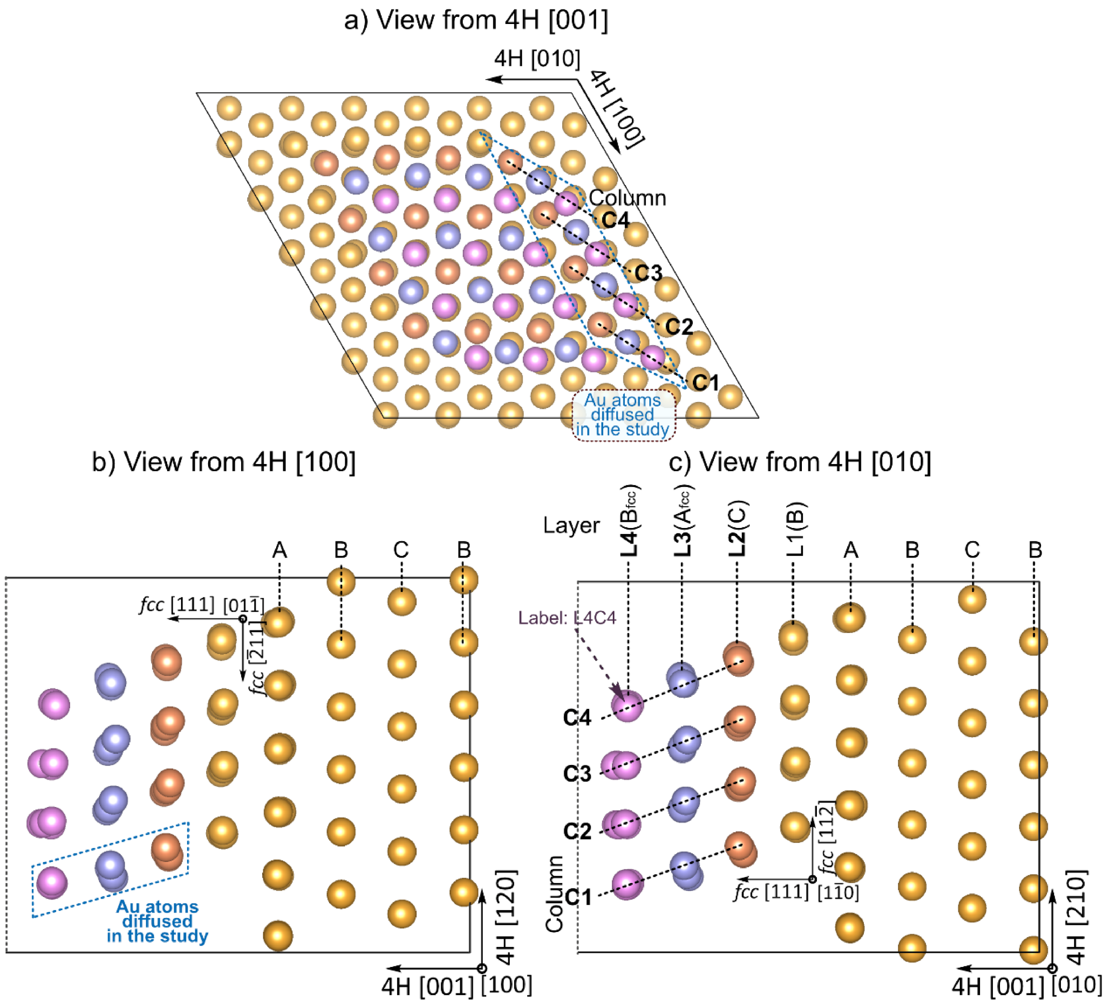

Supplementary Figure 1: **Model of Au diffusion on Au *fcc* pillar**. Calculation model made with a 4-layer Au 4H subatrate surface and a 4-layer Au *fcc* pillar. All atoms in the figure are Au atoms, though for different layer of Au *fcc* pillar different colors are used for clear presentation. The 12 diffused Au atoms in our study are shown in a) & b) and labeled in c)

During the Au diffusion reaction, the reactant Au site and product site are shown in Fig. 5a, and a typical structure change along the reaction is shown in Supplementary Figure 1. When Au atom moves towards 4H surface, it will meet the other Au layer and need overcoming a barrier to pass through, where becomes the transition state.

The transition state is the structure when the diffused Au pass through Layer L2 due to the steric repulsion, as shown in Supplementary Fig. 2. Particularly, for diffused Au atoms from Layer L4, when they pass through L3 and L2, two transition states will be formed. However, according to the CI-NEB calculation shown in Supplementary Figure 3, the first transition state is the dominant TS and rate-determine in this process. For simplification, only this dominant TS is accurately figured

out and discussed.

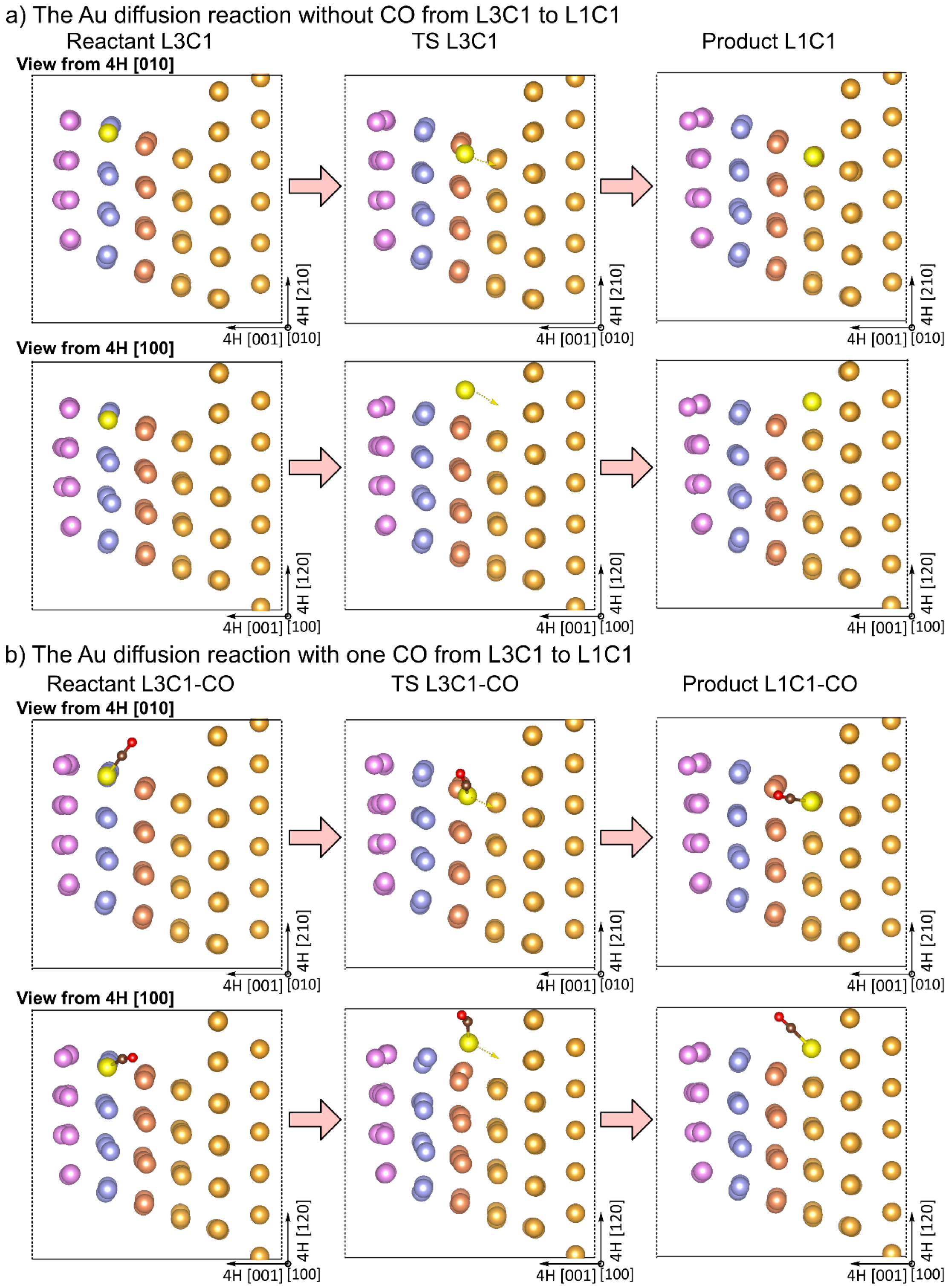

Supplementary Figure 2: Examples on the structure changes. Structure changes along the Au diffusion reaction: from L3C1 to L1C1. The TS is located where the diffused Au meets the Layer L2. a) the case without CO and b) the case with one CO binding to the diffused Au atom. The structures are very similar for the cases of extra electrons and are omitted here.

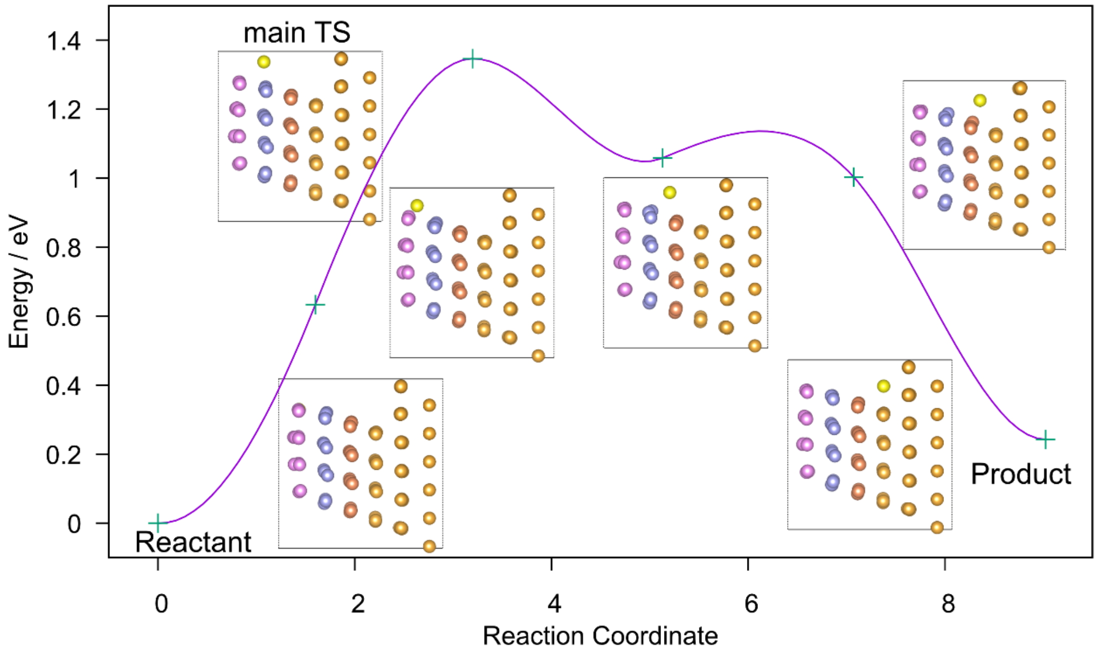

Supplementary Figure 3: A CI-NEB example of Au diffusion reaction from L4C3 to L1C2. Actually, there are two TS when the diffused Au atom passes through the Layer L3 and L2 respectively, but the first TS has the higher relative energy and becomes the dominant TS. Hence the following TS, which is not that important for the reaction profile, is omitted in our calculation.

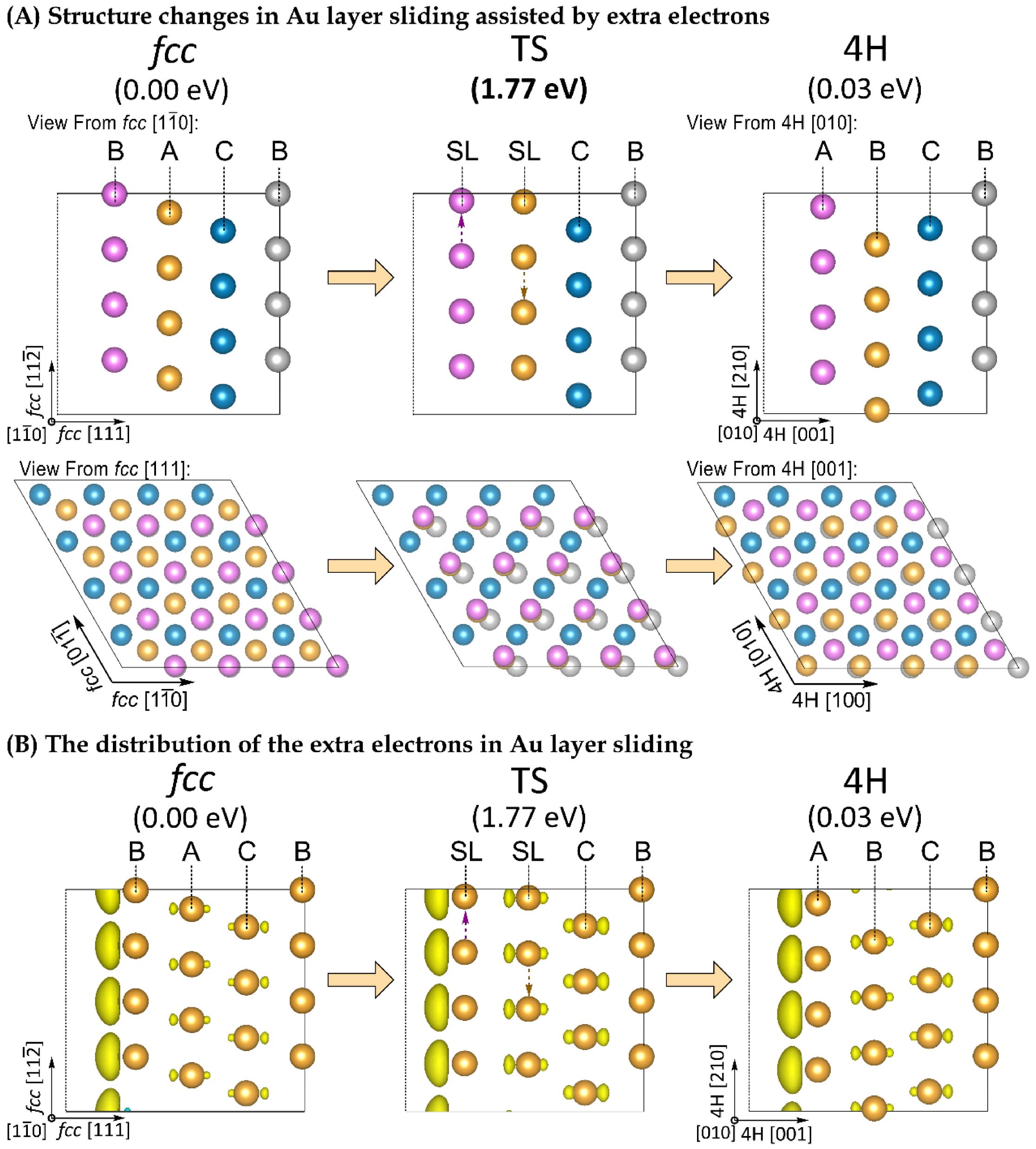

Supplementary Figure 4: **Reaction of *fcc* layer slide.** Au layers slides in the transformation from *fcc* to 4H. a) Structure changes: the view from *fcc* to 4H. The side view is already shown in Fig. 5d. b) Electron distribution of the extra electrons is depicted by yellow areas, which was plotted at the value of ± 0.008 |e|/Å^3.

When CO is not involved in the AIMD calculation, no collapse is observed. Due to the thermal energy in AIMD, the Au atoms on the Au fcc pillar will vibrate around the balance position shown in Fig. 4a, but overall the Au *fcc* pillar still remains. No transformation from *fcc* to 4H is observed in our simulation time.

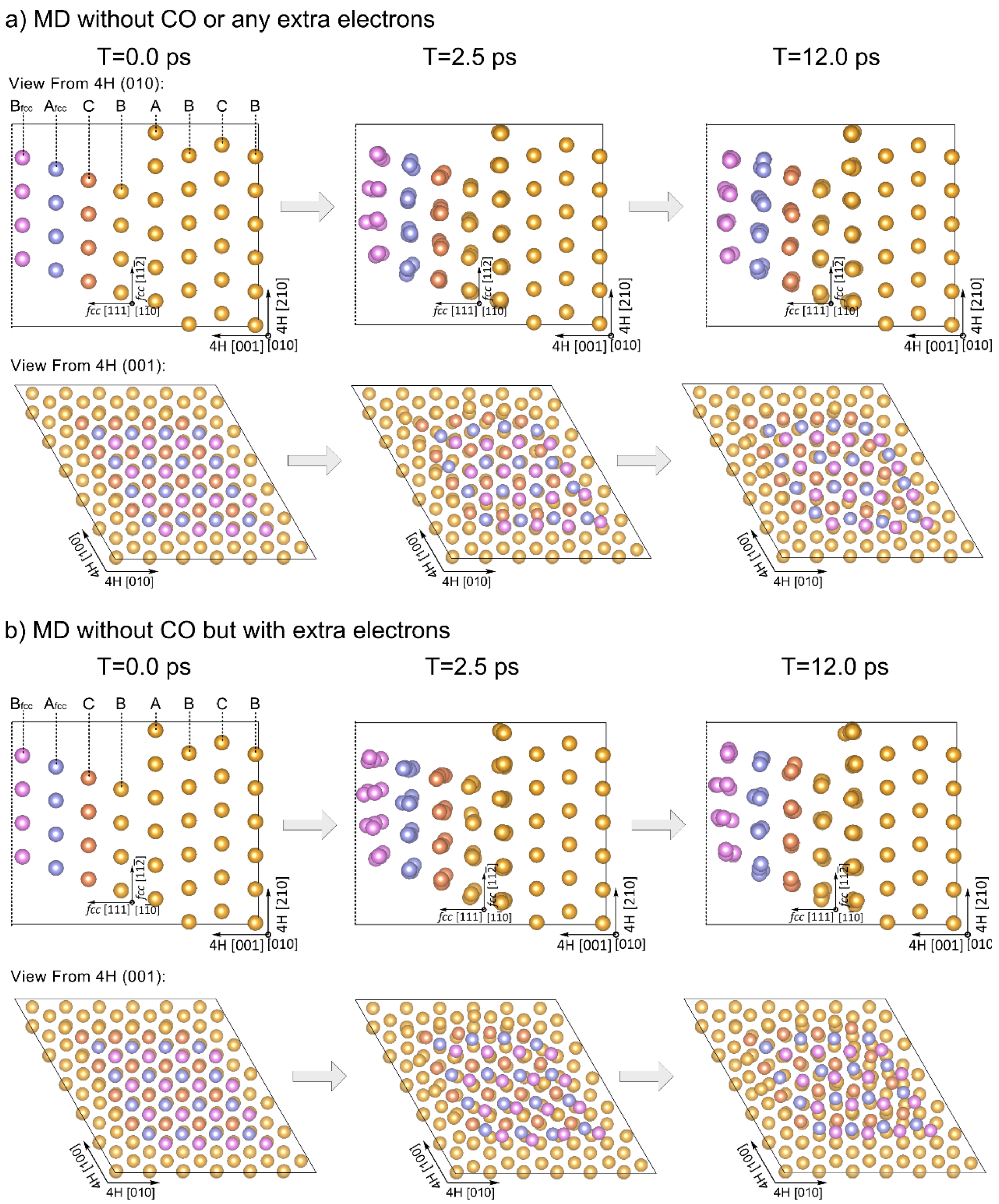

Supplementary Figure 5: Typical trajectory structures in the AIMD. Typical trajectory structures in the AIMD calculation of Au fcc pillar on Au 4H surface without CO. The structures are stable and no collapse is observed. a) neither CO nor extra electrons are added, which is also shown in **Supplementary Movie 5**. b) extra electrons are added without CO, which is also shown in **Supplementary Movie 4**.

In the situation with one CO but no extra electron, as shown in Supplementary Figure 6a, at 1.8 ps, when one Au on L3 is pulled out, another Au on L2 will be diffused to L1. After that, at 3.2 ps, the CO bonded Au will take the place of the diffused Au on L2. The collapse continues and at last (12 ps) the original layer L4 disappears. However, without the help of the extra electrons, the

Au atoms on the 4H substrate still need to be constructed, which is beyond our AIMD simulation time.

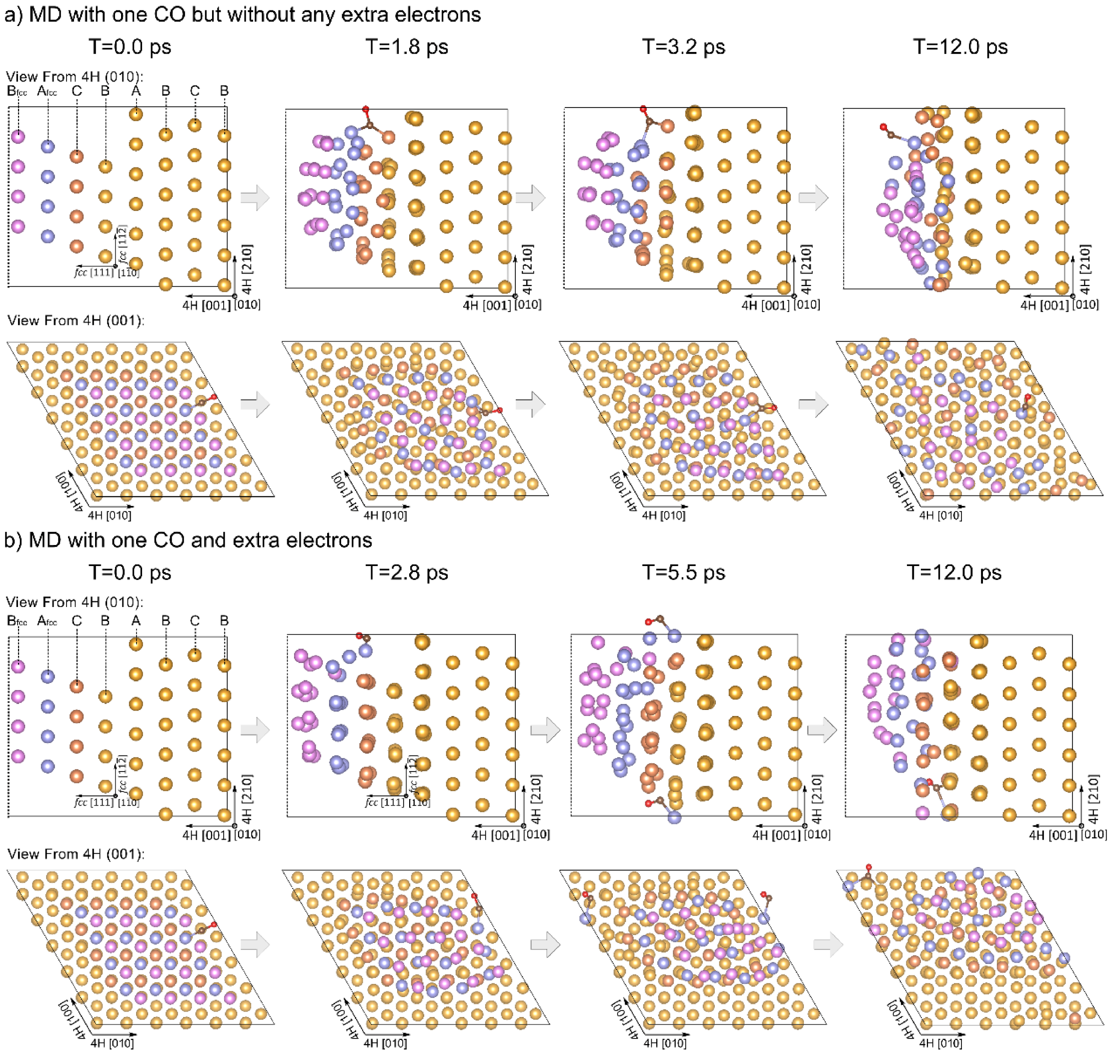

Supplementary Figure 6. Typical trajectory structures in the AIMD calculation of Au fcc pillar on Au 4H surface. a) one CO added but no extra electrons, which is also shown in **Supplementary Movie 3**; b) one CO added with extra electrons, which is also shown in Figure 5 and **Supplementary Movie 2**.

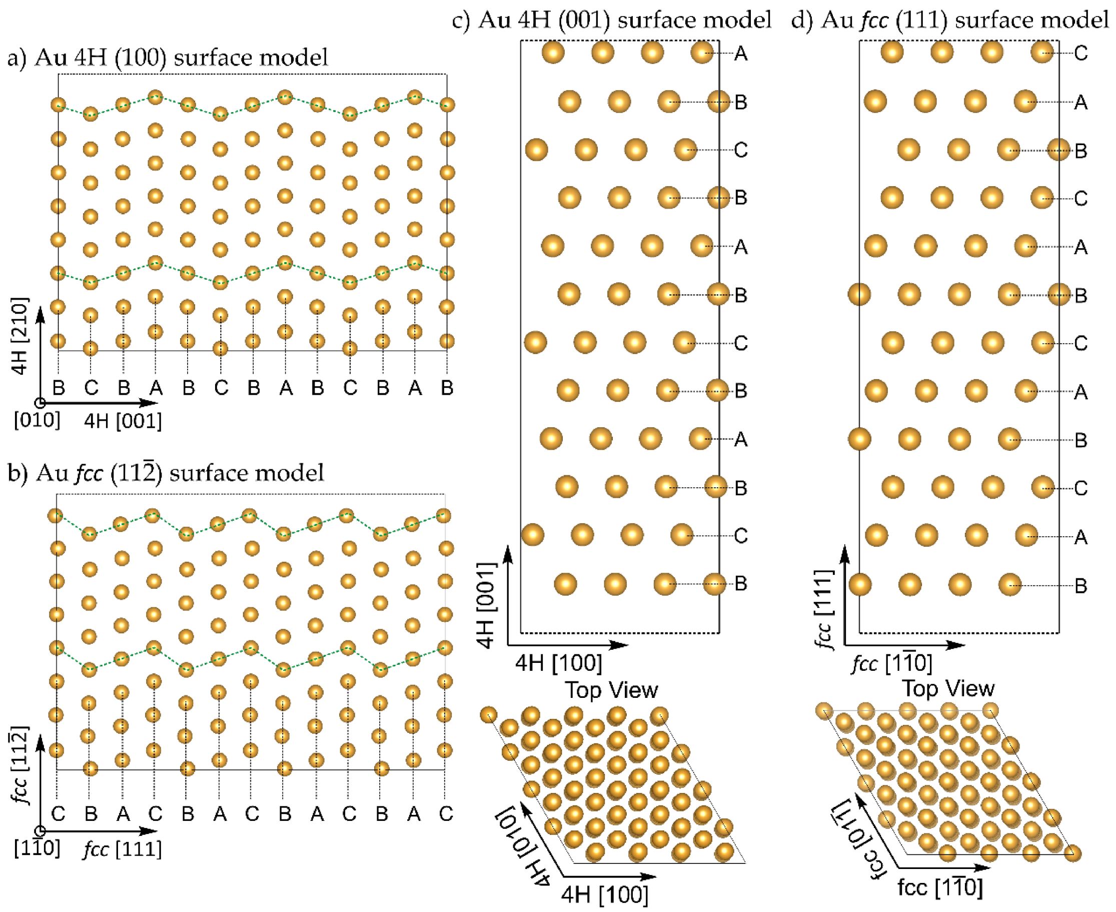

Supplementary Figure 7: Comparison on the surface energies. Models of the surface energy calculation. a) Au 4H (100) surface. 2x3 supercell. b) Au fcc {11$\bar{2}$} surface. 2x4 supercell. (a-b): The eight tortuous layers labeled in green dashed line contain 48 Au atoms per layer. c) Au 4H (001) surface and d) fcc {111} surface. (c-d): 4x4 supercell with 12 layers. Each layer contains 16 Au atoms.

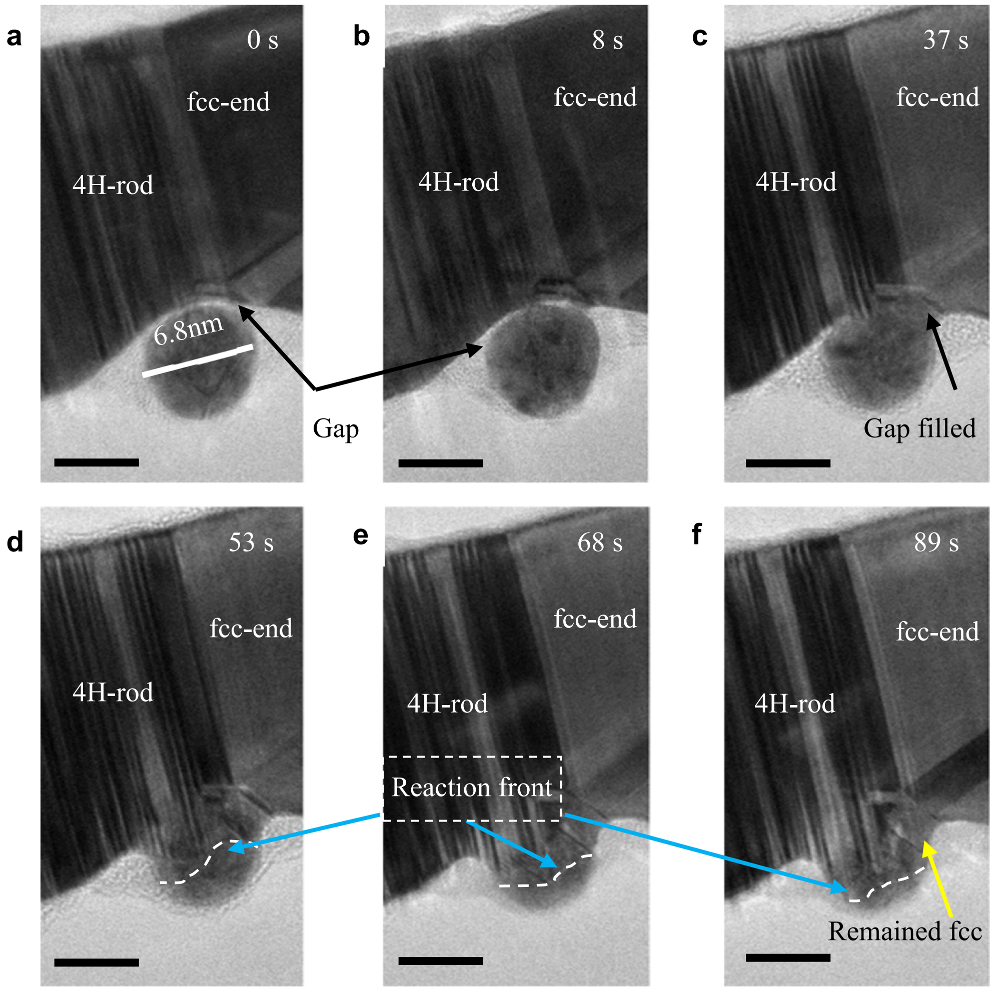

Supplementary Figure 8: Dynamic transformation process from fcc to 4H Au at 80 kV. Phase reaction happened at a lower voltage of 80 kV with electron dose of 500 e Å^{-2} s^{-1} in 1 mbar of CO; the scale bar in panel a can be applied to all panels. The scale bar corresponds to 5.0 nm.

As shown by Supplementary Figure 8, at lower voltage at 80 kV with a dose of 500 e Å^{-2} s^{-1}, we can still observe the phase transformation process clearly. The fcc-Au nanoparticle is about 6.8 nm. Initially, there is a gap between the nanoparticle and 4H-Au rod. After the reaction started, the diffused Au atoms firstly filled the gap. Then, the reaction front push forward to transform most of the fcc nanoparticle to 4H phase within 89 s. Please note that part of the fcc Au nanoparticle is in contact with the fcc-end. The phase transformation is based on an epitaxial growth mechanism, which lowers the overall Gibbs free energy of the system by the elimination the interfaces between the two phases. Therefore, the part close to fcc-end of the rod kept as fcc phase after reaction. The corresponding Supplementary Movie 6 showed this process vividly.

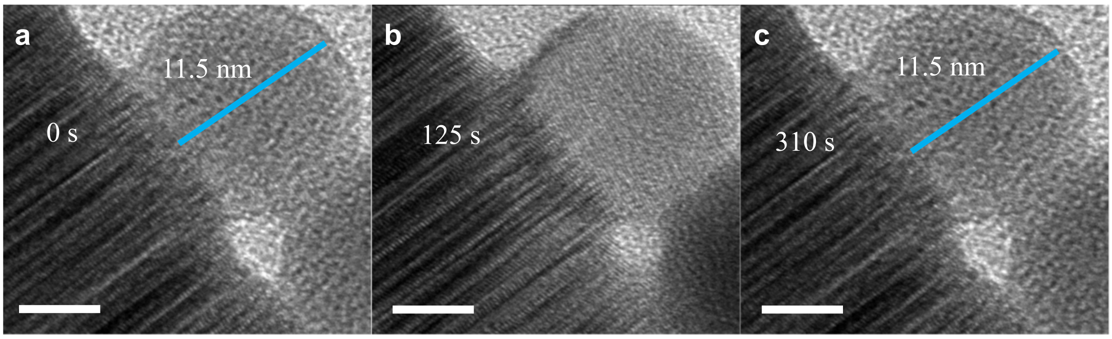

Supplementary Figure 9: Size effect on the phase transformation process at 300kV. Phase transformation halted with bigger *fcc*-Au particles at 300kV with electron dose of 2000 e Å^{-2} s^{-1} in 1mbar of CO. The scale bar corresponds to 5.0 nm.

The phase transformation process halted due to the bigger size of the Au nanoparticle. Therefore, experimentally, we observed that nanoparticles with critical size that is bigger than about 10 nanometers have halted phase transformation. The corresponding Supplementary Movie 7 showed this process vividly.

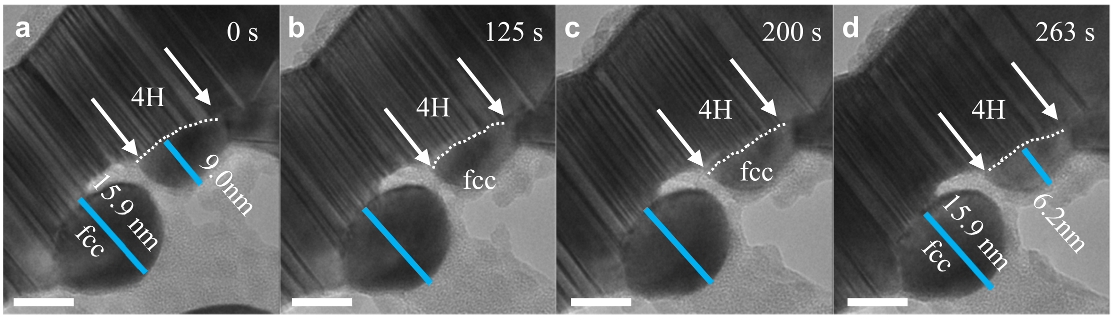

Supplementary Figure 10: Size effect on the phase transformation process at 80 kV. Phase transformation observed for 9 nm nanoparticle; while no phase transformation took place at the bigger Au-nanoparticle of 15.9 nm in the bottom; the dashed white lines label the interface between the 4H rod and fcc-Au nanoparticle with white arrows showing the reaction front progression; imaging condition is 80 kV with electron dose of 500 e Å^{-2} s^{-1} in 1 mbar of CO gas. The scale bar corresponds to 10.0 nm.

Experimentally, we observed the critical size, under which the phase transformation can take place. As shown in Supplementary Figure 10, the small Au-nanoparticle size is around 9 nm and the big Au-nanoparticle size is around 15.9 nm. With the same electron beam dose and voltage, the small particle shows the *fcc*-to-4H phase transformation, while the bigger nanoparticle does not have any changes. The corresponding Supplementary Movie 8 showed this process vividly.

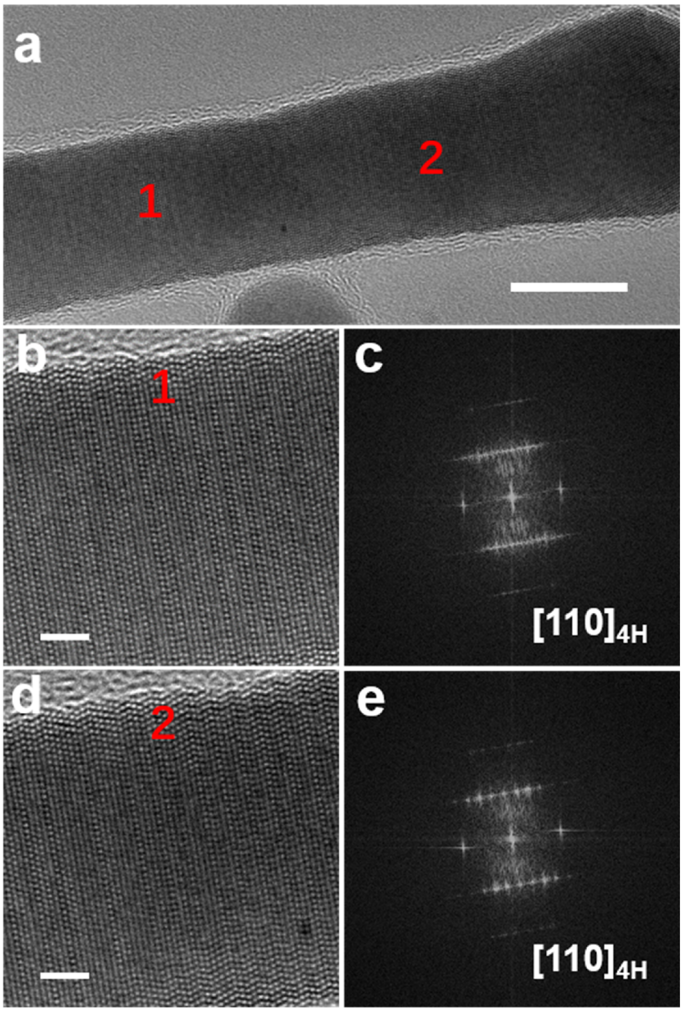

Supplementary Figure 11: HRTEM images and SAED patterns of the 4H ordering nanorod. Additional TEM imaging showing the predominantly 4H symmetry of the gold wire; (a) overall TEM image of the wire (the scale bar corresponds to 10 nm); (b,d) lattice resolution TEM image and corresponding (c,e) FFT of these regions in panel b,d (the scale bar corresponds to 2.0 nm).

Supplementary Tables

Supplementary Table 1. Test on energy cutoff with Gamma K-point. All unit in eV. Reaction on the L3C1 Au diffuse to L1C2 in the 3 Layer Au fcc model.

Cutoff	400	500	600
**Reactant L3C1 Au**	-674.48758	-674.48498	-674.47537
**Product L1C2 Au**	-675.42351	-675.42146	-675.41054
**ΔE**	-0.93593	-0.93648	-0.93517

Supplementary Table 2. Test on K-points with the energy cutoff of 400 eV. All K-points are sampled from Gamma point. All unit in eV. Reaction on the L3C1 Au diffuse to L1C2 in the 3 Layer Au fcc model.

K-points	1×1×1	1×2×2	1×3×3	1×4×4
**Reactant L3C1 Au**	-674.48758	-678.01505	-679.36425	-679.17735
**Product L1C2 Au**	-675.42351	-678.96354	-680.29653	-680.11751
**ΔE**	-0.93593	-0.94849	-0.93228	-0.94017

Supplementary Table 3: Active energy (E_a) and reaction energy (ΔE) of different diffused Au atoms. Active energy (E_a) and reaction energy (ΔE) of different diffused Au atoms. The model is shown in Supplementary Figs. 1 and 2 and the label is shown in Figure 4a. All energy unit in eV.

Reactant Au site	**L4C1**	**L4C2**	**L4C3**	**L4C4**
Product Au site	L1C1	L1C1	L1C2	L1C3
$E_{a(dominant)}$	0.71	1.08	1.35	1.03
ΔE	-0.90	-0.26	0.24	-0.17
Reactant Au site	**L3C1**	**L3C2**	**L3C3**	**L3C4**
Product Au site	L1C1	L1C1	L1C2	L1C3
E_a	1.13	1.14	1.40	1.37
ΔE	-0.24	0.13	0.34	0.20
Reactant Au site	**L2C1**	**L2C2**	**L2C3**	**L2C4**
Product Au site	near L1C2	L1C1	L1C2	L1C3
E_a	0.84	0.91	0.78	0.80
ΔE	0.53	0.59	0.45	0.27
Reaction with extra electrons:				
Reactant Au site	**L4C1**	**L4C2**	**L4C3**	**L4C4**
Product Au site	L1C1	L1C1	L1C2	L1C3
$E_{a(dominant)}$	0.66	1.00	1.36	0.91
ΔE	-0.85	-0.32	0.22	-0.16
Reactant Au site	**L3C1**	**L3C2**	**L3C3**	**L3C4**
Product Au site	L1C1	L1C1	L1C2	L1C3
E_a	1.00	1.04	1.26	1.18
ΔE	-0.32	0.11	0.34	0.25
Reactant Au site	**L2C1**	**L2C2**	**L2C3**	**L2C4**
Product Au site	near L1C2	L1C1	L1C2	L1C3
E_a	0.96	0.89	0.82	0.78
ΔE	0.28	0.48	0.44	0.30

Reaction with one CO:				
Reactant Au site	**L4C1**	**L4C2**	**L4C3**	**L4C4**
Product Au site	L1C1	L1C1	L1C2	L1C3
$E_{CO\_binding}$	-0.24	0.11	0.09	0.09
$E_{a(dominant)}$	0.37	1.06	0.76	0.67
ΔE	-0.77	-0.48	0.08	-0.02
Reactant Au site	**L3C1**	**L3C2**	**L3C3**	**L3C4**
Product Au site	L1C1	L1C1	L1C2	L1C3
$E_{CO\_binding}$	0.07	0.20	0.24	0.09
E_a	0.53	0.47	0.63	0.66
ΔE	-0.39	-0.17	0.02	0.08
Reactant Au site	**L2C1**	**L2C2**	**L2C3**	**L2C4**
Product Au site	L1C1	L1C1	L1C2	L1C3
$E_{CO\_binding}$	0.00(set to 0)	0.39	0.37	-0.01
E_a	0.46	0.31	0.05	0.39
ΔE	0.38	0.10	0.04	0.23

Supplementary Table 4. Estimated temperature rise due to the beam irradiation effect at varies conditions. The beam heating effect on the temperature rise at varies voltages and dose rates.

	80 kV	300 kV	
Dose rates (e $Å^{-2}$ s^{-1})	500	1000	4000
Cross section barns	2.0×10^{6}	1.47×10^{5}	1.47×10^{5}
Fraction scattered	2.4×10^{-4}	1.73×10^{-5}	1.73×10^{-5}
Maximum transferred energy (eV)	<1.22	4.32	4.32
Temperature rise (ΔT/K)	90	45	180

Supplementary Discussion

Comparison on the surface energies of Au $\{11\bar{2}\}_{fcc}$, $(100)_{4H}$, $\{111\}_{fcc}$ and $(001)_{4H}$.

As shown in Fig. 2(j-l), the {111} surfaces of fcc-nanoparticles are aligned with the (004) surfaces of 4H rod. However, with the lattice mismatch and high surface energy, this interface is neither perfect 4H surface nor *fcc* surface, which contains rearrangements of Au atoms and defects. The interface surface between the sintered Au *fcc* nanoparticle and 4H nanorod substrate is mainly perpendicular to $[001]_{4H}$ direction. A rough but representative atomic model involving the interface between $\{11\bar{2}\}_{fcc}$ and $(100)_{4H}$ surface is shown in Fig 6(c). The surface energies of $\{11\bar{2}\}_{fcc}$, $(100)_{4H}$, $\{111\}_{fcc}$ and $(001)_{4H}$ are calculated as follow:

$$E_{\text{surface}} = \frac{E_{\text{model}} - n\,E_{\text{perAu}}}{2A} \qquad (1)$$

In which the E_{surface} is the surface energy. E_{model} is the calculated energy of the model in Supplementary Fig. 7. n is the total number of Au atoms in each model. E_{perAu} is the formation energy for bulky Au crystal, which is calculated to be -3.85856 eV per Au for 4H packing and -3.86121 eV per Au for fcc packing. A is the area of the surface, which is calculated by the dot product of the two cell vectors in the surface plane.

Surface Energy calculated by the model in Supplementary Fig. 7:

Au 4H (100): 1.395 J m^{-2}

Au fcc $\{11\bar{2}\}$: 1.510 J m^{-2}

Au 4H (001): 1.306 J m^{-2}

Au fcc {111}: 1.301 J m^{-2}

In these four surfaces, Au fcc {111} and Au 4H (001) are both stable surface with relatively low surface energy.

Au 4H (100) and Au fcc $\{11\bar{2}\}$ are less-stable surfaces.

Au fcc $\{11\bar{2}\}$ is considerably less stable than Au 4H (100).

For the model in Supplementary Fig. 7(a) and (b), with the area of 332.3 $Å^2$, the surface energy difference is 2.387eV×2=4.774 eV for two surfaces.

Considering for bulky crystal, *fcc* is more stable than 4H. With 48 atoms in each tortuous layer, each layer contributes the stacking energy difference of 0.1271eV.

This implies at least 38 tortuous layers is need to make fcc $\{11\bar{2}\}$ surface more favorable than 4H (100) surface, although they share similar packing topology. This corresponds to around 9.5 nm.

Supplementary Method

Estimated temperature rise due to the beam irradiation effect. The beam electron has to penetrate close to the nucleus and be effectively assisted the face slide, thus the maximum transferable Kinetic energy at 80 kV, 200 kV and 300 kV estimated, it is less than 1.22 eV, 2.67 eV and 4.32 eV, respectively.

Multiplying the number of targets per square meter times the cross-section for each target gives you the fraction of the area that is covered by target and therefore the fraction of the beam that would be scattered. [1]

$$\text{fraction scattered}=\frac{R_s}{R_i}=\frac{N_A L\rho\sigma}{A10^{-3}\text{kg}} \qquad (2)$$

R_i: incident rate, R_s: Scattered rate, L= Au nanorod thickness (20×10^{-9} m), ρ=gold density (19300 kg m^{-3}), A=atomic mass, N_A=Avogadro's number=6.02×10^{23} mol^{-1}, σ: cross sections for the inelastic scattering processes, for gold atoms it is in the order of $\sim10^5$ magnitude. Bringing these parameters into above equation, and then fraction scattered valve can be worked out which provides a theoretical basis for determine the probability of electrons being scattered by the Au nanorod. The result of the calculation is about 1.2×10^{-7}. That means for an incident rate of 10^5 e s^{-1} nm^{-2}, an average of 1.2 $\times10^{-2}$ e s^{-1} nm^{-2} would be expected to be scattered.

The Au nanorod diameter=20 nm, length=200 nm, volume ≈ 60000 nm^3, 1 nm^3~ 64 Au atoms, the total number of Au atoms ≈ 60000×64 =3840000. According to the formula E=1.5 k_b T, the maximum transferred energy per second=48 eV (k_b is the Boltzmann constant).

The corresponding Supplementary Table 4 showed these results.

Supplementary References

1 Williams, D. B. & Carter, C. B. in *Transmission electron microscopy* 3-17 (Springer, 1996).
