# Supplementary material for: Gas-assisted transformation of gold from fcc to the metastable 4H phase
Source: Nat Commun. 2020 Jan 28;11:552. doi: 10.1038/s41467-019-14212-z (PMC6987310; doi:10.1038/s41467-019-14212-z)
Supplement: Supplementary file 2 — Description of Additional Supplementary Files [file 41467_2019_14212_MOESM2_ESM.pdf]

**Description of Additional Supplementary Files**

File Name: Supplementary Movie 1
Description: Dynamic transformation process from fcc to 4H Au at 300 kV with 1.0 mbar CO and dose rate of 2000 e $Å^{-2}$ $s^{-1}$

File Name: Supplementary Movie 2
Description: AIMD with 1 CO and extra electrons

File Name: Supplementary Movie 3
Description: AIMD with 1 CO but no extra electrons

File Name: Supplementary Movie 4
Description: AIMD with extra electrons but no CO.

File Name: Supplementary Movie 5
Description: AIMD with neither extra electrons nor CO

File Name: Supplementary Movie 6
Description: Dynamic transformation process from fcc to 4H Au at 80 kV with 1.0 mbar CO and dose rate of 500 e $Å^{-2}$ $s^{-1}$.

File Name: Supplementary Movie 7
Description: Size effect on the phase transformation process at 300 kV with 1.0 mbar CO and dose rate of 2000 e $Å^{-2}$ $s^{-1}$.

File Name: Supplementary Movie 8
Description: Size effect of the phase transformation process at 80 kV with 1.0 mbar CO and dose rate of 500 e $Å^{-2}$ $s^{-1}$.
