# Supplementary figures and images for: Gas-assisted transformation of gold from fcc to the metastable 4H phase
Source: Nat Commun. 2020 Jan 28;11:552. doi: 10.1038/s41467-019-14212-z (PMC6987310; doi:10.1038/s41467-019-14212-z)

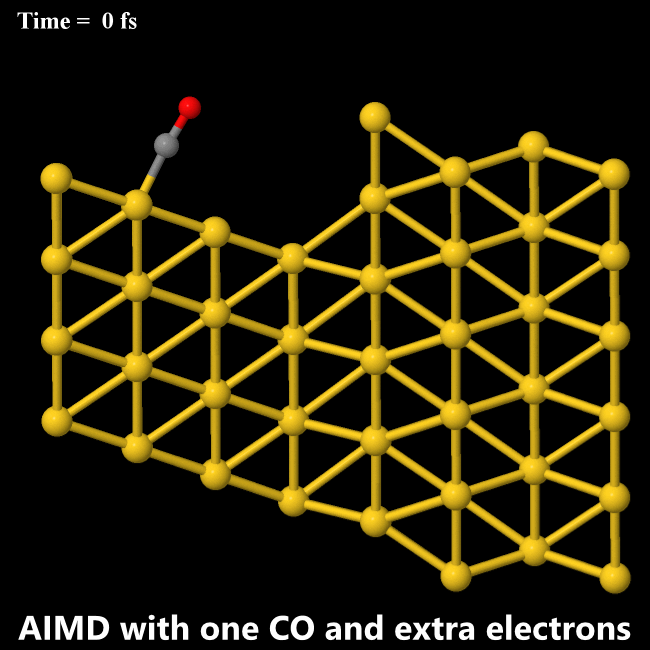

Supplement: Supplementary file 4 — Supplementary Movie 2 [file 41467_2019_14212_MOESM4_ESM.gif]

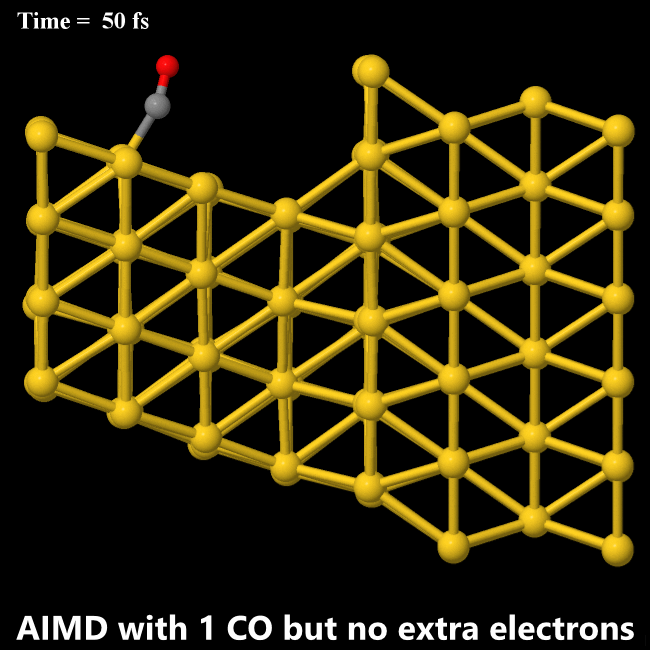

Supplement: Supplementary file 5 — Supplementary Movie 3 [file 41467_2019_14212_MOESM5_ESM.gif]

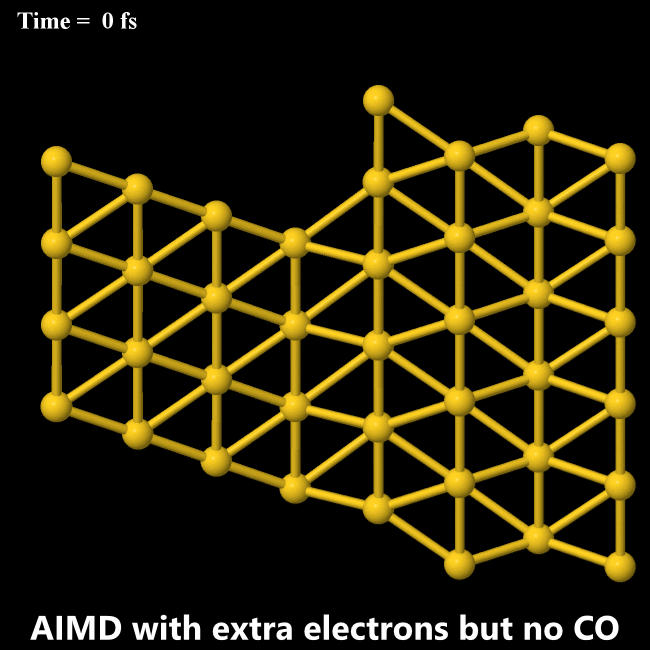

Supplement: Supplementary file 6 — Supplementary Movie 4 [file 41467_2019_14212_MOESM6_ESM.gif]

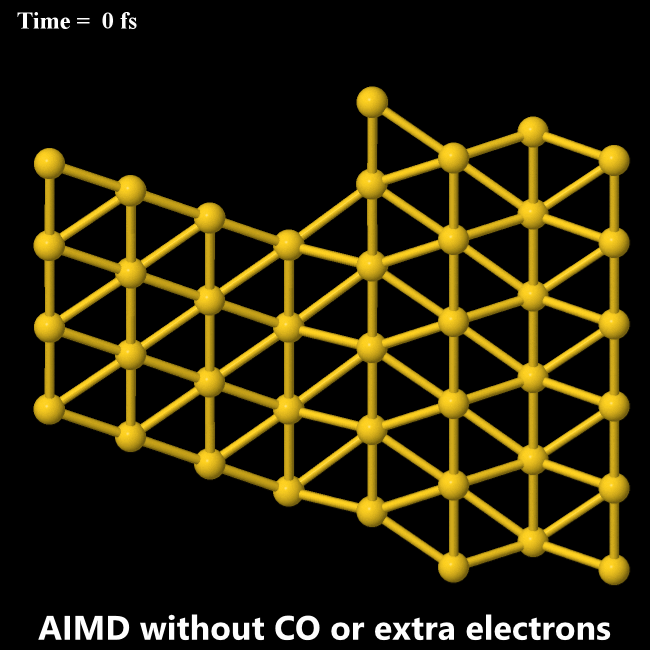

Supplement: Supplementary file 7 — Supplementary Movie 5 [file 41467_2019_14212_MOESM7_ESM.gif]
